# Supplementary material for: Maternal complications in pregnancy and childbirth for women with epilepsy: Time trends in a nationwide cohort
Source: PLoS One. 2019 Nov 25;14(11):e0225334. doi: 10.1371/journal.pone.0225334 (PMC6876881; doi:10.1371/journal.pone.0225334)
Supplement: S1 Fig — (DOCX) [file pone.0225334.s001.docx]

**Figure S1. Percentage of WWE with the four most common AEDs in monotherapy per 100 WWE with any AEDs during 1999-2016.**
