# Supplementary material for: Maternal complications in pregnancy and childbirth for women with epilepsy: Time trends in a nationwide cohort
Source: PLoS One. 2019 Nov 25;14(11):e0225334. doi: 10.1371/journal.pone.0225334 (PMC6876881; doi:10.1371/journal.pone.0225334)
Supplement: S1 Table — (DOCX) [file pone.0225334.s002.docx]

| **Table S1. Total complications in first pregnancies of women with epilepsy who used the four most common antiepileptic drugs in monotherapy compared to women without epilepsy 1999-2016** | | | | | | | | | |
| --- | --- | --- | --- | --- | --- | --- | --- | --- | --- |
|  | | | | | | | | | |
|  | **Women without epilepsy** | **Lamotrigine** | | **Carbamazepine** | | **Levetiracetam** | | **Valproat** | |
|  | **N=423 270** | **N=437** | | **N=243** | | **N=118** | | **N=130** | |
|  |  |  | |  | |  | |  | |
|  |  | **N (%)** | **aOR** | **N (%)** | **aOR** | **N (%)** | **aOR** | **N (%)** | **aOR** |
|  |  |  |  |  |  |  |  |  |  |
| **Any hypertensive disorder** | 29 231 (6.9) | 31 (7.1) | 1.01 (0.70-1.46) | 29 (11.9) | 1.73 (1.17-2.56) | 8 (6.8) | 0.94 (0.46-1.93) | 14 (10.8) | 1.73 (0.99-3.02) |
|  |  |  |  |  |  |  |  |  |  |
| **Mild preeclampsia** | 12 445 (2.9) | 10 (2.3) | 0.74 (0.39-1.38) | 20 (8.2) | 2.85 (1.80-4.51) | 5 (4.2) | 1.36 (0.55-3.33) | 8 (6.2) | 2.20 (1.07-4.50) |
|  |  |  |  |  |  |  |  |  |  |
| **Severe preeclampsia** | 7875 (1.9) | 10 (2.3) | 1.19 (0.64-2.24) | 5 (2.1) | 1.02 (0.42-2.49) | 2 (1.9) | 0.84 (0.21-3.43) | 3 (2.3) | 1.36 (0.43-4.29) |
|  |  |  |  |  |  |  |  |  |  |
| **Induction of labour** | 53 203 (14.6) | 88 (22.7) | 1.55 (1.22-1.98) | 31 (15.8) | 0.99 (0.67-1.47) | 36 (33.6) | 2.67 (1.78-4.02) | 22 (20.8) | 1.54 (0.95-2.45) |
|  |  |  |  |  |  |  |  |  |  |
| **Emergency cesarean section*** | 56 307 (13.3) | 67 (15.3) | 1.13 (0.86-1.47) | 42 (17.3) | 1.22 (0.87-1.72) | 15 (12.7) | 0.82 (0.47-1.42) | 19 (14.6) | 1.16 (0.71-1.90) |
|  |  |  |  |  |  |  |  |  |  |
| **Elective cesarean section** | 15 572 (3.7) | 29 (6.6) | 1.92 (1.31-2.81) | 24 (9.9) | 2.47 (1.61-3.79) | 9 (7.6) | 2.27 (1.14-4.50) | 9 (6.9) | 2.22 (1.12-4.40) |
|  |  |  |  |  |  |  |  |  |  |
| **Preterm birth** | 19 725 (6.0) | 17 (5.7) | 0.95 (0.58-1.56) | 18 (10.6) | 1.74 (1.06-2.86) | 5 (7.2) | 1.33 (0.53-3.33) | 4 (4.5) | 0.83 (0.30-2.26) |
|  |  |  |  |  |  |  |  |  |  |
| **Bleeding in pregnancy** | 20 989 (5.0) | 30 (6.9) | 1.15 (0.79-1.68) | 9 (3.7) | 0.68 (0.35-1.33) | 5 (4.2) | 0.71 (0.29-1.75) | 4 (3.1) | 0.53 (0.19-1.43) |
|  |  |  |  |  |  |  |  |  |  |
| **Postpartum hemorrhage** | 6963 (2.0) | 11 (3.2) | 1.55 (0.85-2.83) | 5 (2.8) | 1.34 (0.55-3.26) | 3 (3.2) | 1.50 (0.48-4.76) | 5 (4.9) | 2.55 (1.04-6.27) |
|  |  |  |  |  |  |  |  |  |  |
| **Small for gestational age** | 7788 (1.8) | 8 (1.8) | 1.09 (0.54-2.19) | 7 (2.9) | 1.52 (0.71-3.22) | 4 (3.4) | 2.07 (0.76-5.61) | 3 (2.3) | 1.34 (0.43-4.21) |
|  |  |  |  |  |  |  |  |  |  |
| **Epidural analgesia** | 181 531 (42.9) | 254 (58.1) | 1.73 (1.43-2.09) | 120 (49.4) | 1.30 (1.01-1.67) | 69 (58.4) | 1.71 (1.18-2.48) | 73 (56.2) | 1.61 (1.14-2.28) |
|  | | | | | | | | | |

aOR = Adjusted odds ratio

All outcomes adjusted for: maternal age, smoking, folic acid supplementation, chronic diseases.

*Also adjusted for induction of labour
