# Supplementary material for: Maternal complications in pregnancy and childbirth for women with epilepsy: Time trends in a nationwide cohort
Source: PLoS One. 2019 Nov 25;14(11):e0225334. doi: 10.1371/journal.pone.0225334 (PMC6876881; doi:10.1371/journal.pone.0225334)
Supplement: S2 Table — (DOCX) [file pone.0225334.s003.docx]

| **Table S2. Maternal characteristics of 426 347 first births for women with epilepsy and for women without epilepsy 1999-2016.** | | | |
| --- | --- | --- | --- |
|  | | | |
|  | **WOMEN WITHOUT EPILEPSY** | **EPILEPSY** |  |
|  |  |  |  |
|  | **423 270** | **3077** |  |
|  | **N (%)** | **N (%)** | **Value of P** |
|  |  |  |  |
| **Maternal age (mean years)** | 28.0 | 27.7 | <0.005 |
|  |  |  |  |
| **Unemployed** | 58 806 (13.9) | 554 (18.0) | <0.005 |
|  |  |  |  |
| **Marital status (single vs. married or cohabitant)** | 379 783 (89.7) | 2657 (86.4) | <0.005 |
|  |  |  |  |
| **Gestational age (mean days)** | 278.9 | 277.7 | <0.005 |
|  |  |  |  |
| **Birth weight (mean grams)** | 3445 | 3407 | <0.005 |
|  |  |  |  |
| **Body Mass Index (mean kg/m2) *** | 23.9 | 24.5 | <0.005 |
|  |  |  |  |
| **Smoking (yes)** | 55 866 (13.2) | 593 (19.3) | <0.005 |
|  |  |  |  |
| **Other chronic disease **** | 8002 (1.9) | 217 (7.1) | <0.005 |
|  |  |  |  |
| **Folic acid supplementation** | 245 813 (58.1) | 2038 (66.2) | <0.005 |
|  |  |  |  |
| **Native Norwegians** | 331 106 (78.2) | 2632 (85.5) | <0.005 |
|  |  |  |  |
| **Assisted reproduction** | 14 338 (3.4) | 99 (3.2) | 0.648 |
|  |  |  |  |
| **Hospital stay (mean days)** | 3.64 | 3.94 | <0.005 |
|  | | | |

*Registered from 2006

** Hypertension, Kidney disease, Diabetes
