# Supplementary material for: Maternal complications in pregnancy and childbirth for women with epilepsy: Time trends in a nationwide cohort
Source: PLoS One. 2019 Nov 25;14(11):e0225334. doi: 10.1371/journal.pone.0225334 (PMC6876881; doi:10.1371/journal.pone.0225334)
Supplement: Attachment — Submitted filename: Respons to Reviewers.docx [file pone.0225334.s004.docx]

To The Editor

PLOS ONE	October 2019

Dear sir,

Attached is our revised version of the submitted manuscript “Maternal complications in pregnancy and childbirth for women with epilepsy: time trends in a nationwide cohort” (PONE-D-19-16520), which we hope would be acceptable for publication in PLOS ONE.

We thank the editors and reviewers for their constructive comments and suggestions. Below is our point-by-point response to the posed questions and comments.

**Reviewer #1:**

1. *My major concern is the chosen methodology. Authors report significant changes in maternal characteristics and study outcomes both in WWE and in reference population. Also, there has been fundamental change in the pattern of AED use. Still, year of delivery/triennial period of delivery is not included in the adjusted regression models. Not even in the sensitivity analyses. In my opinion, time-varying elements have not been adequately addressed. Authors should adjust their risk estimates for year of delivery, or they could even consider alternative approaches such as age-period-cohort analysis. Another limitation is the BMI which was not possible to account for. Authors did not find any change in mean BMI 2006-2016 which seem surprising as obesity has increased during the last decades in many western countries. Moreover, it does not guarantee that 1996 and 2016 are comparable even though there would not have been change between 2006 and 2016. BMI is an important factor in terms of obstetric complications and could be tackled e.g. by imputation. It is not clear why country of origin is not among adjusted covariates as there is significant difference in proportions among WWE and reference population. Also, the proportion of immigrants has increased dramatically over the study period for which it is also an important factor for time trend analysis.*

**Response:**

Year of delivery (triennial) was included in the final regression models for the examination of both time-trends and as part of the interaction analyses. These are the main analyses in our study. Regression analyses for time-trends and interaction with time are therefore both adjusted for other covariates. Furthermore, the analyses were repeated in stratified analyses for every stratum of risk factors in the sensitivity analyses in order to gain knowledge regarding how total AEDs, specific AEDs, and covariates independently influence outcomes over time. In contrast, our results shown in the tables represent the total examined population, serve as background data, and without the effect of time included. We examined time as a possible effect-modifier in our study. Therefore, we found it more appropriate to show the results from time-trend and time-interaction analyses separate from the overall tables. We acknowledge the unclear description of the statistical analyses. We have tried to clarify this description in the revised manuscript (page 6, line 190-201 in manuscript with tracked changes). See also response to comment 2, below.

We acknowledge BMI as an important variable for obstetric complications and should be considered as a potential confounder. There are however, limitations in the BMI-variable in the MBRN, including incompleteness. When we found little evidence that adjustment for the BMI-variable altered the results, we decided not to include the problematic BMI-variable in the main regression models. We have revised and highlighted these considerations and limitations in the manuscript (page 10, line 308-309 and page 14, line 438-445). The lack of change of BMI in our study was not expected. We agree that this lack of change is not necessarily synonymous with equivalence. This has been specified in the discussion of our revised manuscript (page 14, line 438-445).

As suggested by the reviewer, we have now analyzed all outcomes with maternal nationality as a confounding factor included in the logistic regression models. This inclusion had minimal impact on effect measures and changed estimates only at the level of the second decimal. The impact on effect measures was smaller than 5 % for all outcomes, including time trends and interaction analyses. Maternal nationality was therefore not included in the final regression models. However, it is now included in the description of possible adjustment variables in the methods section (page 6, line 187).

2. *Another, somewhat related, major issue is that stratification-based sensitivity analyses do not handle the fact that maternal characteristics were not stable over time. Authors conclude that increase in relative risk of severe pre-eclampsia for WWE was not associated with other covariates. Only when the combination of covariates was considered, relative risk (RR) of severe pre-eclampsia in WWE was reversed. Variables included in the sensitivity analyses are those associated with socioeconomic status and these typically cumulate to same individuals. Therefore, one might similarly argue that combination should be in the final model and draw the conclusion that the risk of severe pre-eclampsia is not increased. In my opinion, authors make rather strong interpretation on lines 244-245. There is some bizarre ad hoc analysis on lines 297-305. Induction and elective c-section before term is due to life-threatening circumstances either for the mother or for the child. Thus, this is selected and biased population. I do not understand what is the aim and rationale of this analysis.*

**Response:**

Sensitivity analyses were similar to the main analyses, but repeated in stratum of sub-groups. Thus, sensitivity analyses also include possible effects of time. We agree that some of the stratified variables in the sensitivity analyses are associated with socioeconomic status. The reviewer suggests combining the variables. We did this as a sub-analysis to explore influences on severe preeclampsia in WWE only and not for other outcomes. For this outcome, we have also included the antecedent analysis of the individual variables (i.e. the original sensitivity analyses). We have now specified the results and discussion regarding this sub analysis (page 8, line 244-245). We have also improved the description of the analyses in the methods section (page 6, line 190-201). Only a selected set of a high number of sensitivity analyses have been reported in the results of the manuscript.

We have now rephrased the lines 244-245 referred to by the reviewer.

Preterm birth was increased in WWE (and in WWE with carbamazepine). Therefore, we performed further analyses in order to gain knowledge and explore morbidity among all preterm births. Severe complications were increased in women who gave birth preterm (e.g. severe preeclampsia) (1, 2). Our principal analysis of preterm births was intended to explore spontaneous births; therefore excluding births with induction of labour and elective cesarean section. However, in the sub-analyses these variables were included. We acknowledge the fact that these interventions indeed are performed with increased frequency in births with severe complications. This is why we include them in the sub-analysis. We have now rephrased the reasoning behind and description of these analyses in the manuscript (page 10, line 295-300).

3. *Some of the results seem controversial, e.g. increased risk for severe pre-eclampsia but decreased risk for mild pre-eclampsia in WWE. Authors haven´t paid much attention to this in the discussion. They say that their study lacks data on seizure activity and that never AEDs are associated with higher seizure frequencies in pregnancy. Could there be misclassification of seizure and severe pre-eclampsia/eclampsia so that epileptic seizures are registered as eclampsia? Could this explain why we don´t observe the same reduction of severe pre-eclampsia in WWE than in the reference population? Could there be verification bias due to more intensive surveillance for WWE women? In general, is there any biological explanation why risk pattern in WWE would be different for severe and mild eclampsia? There are 11 different outcomes and four individual AEDs. It is very likely that some of the results are significant only by chance because of the multiple testing for which P-values have not been corrected for.*

Response:

We acknowledge the apparent contradiction in some of our results. We appreciate the remark and have now elaborated our discussion on this point (page 11-12, line 354-360).

The reviewer propose a possible misclassification of true epileptic seizures as seizures due to eclampsia. If epileptic seizures were misclassified as eclampsia we would have expected to find significant associations between eclampsia and WWE. There was no such association. The number with eclampsia in WWE were lower than expected if epileptic seizures were misclassified. Outcomes for eclampsia in WWE have previously been published on a subgroup of this population (3).

Ascertainment bias is not expected to influence severe conditions. Several severe conditions have been validated in the MBRN with acceptable registrations and positive predictive values mostly exceeding 90 % (4-10). Differences in registration between WWE and women without epilepsy might be more relevant for variables that are more trivial. Therefore, a possible ascertainment bias for mild preeclampsia cannot completely be excluded. We have expanded the discussion regarding possible ascertainment bias in the revised manuscript (page 13, line 417-421).

We have chosen not to correct p-values for random errors by multiple testing. Since our focus is on estimation rather than formal hypothesis testing, we have removed p-values and put more emphasis on estimates and confidence intervals. P-values are important, however, in the evaluation of interactions and changes over time.

4. *There is quite a lot of underlying assumptions/interpretations that are not written out but assumed to be self-explanatory which they are not. E.g. it is not clear what WWE were excluded from analyses (lines 134-135). Those that used named four AEDs as polytherapy? Does any use only include only these four or any AED as monotherapy? More ambiguous text is listed under the minor concerns.*

**Response:**

We have analyzed: 1) All WWE regardless of treatment or not, 2) WWE without AED treatment, 3) All WWE with any AED treatment (any monotherapy and all combinations of polytherapy), 4) WWE with specified AEDs in monotherapy (lamotrigine, levetiracetam, valproate or carbamazepine). We have rephrased the description of sub-categories of WWE in the revised manuscript in line with the suggestions from the referee (page 4, line 130-134 and page 6, line 176-178).

5. *Minor concerns:*
   a) *-increased focus on women with epilepsy? L70-71 What do you mean by increased focus?*
   b) *-The concept of validity on lines 129-132. Two dimensions of data quality are completeness and accuracy. It is not clear how valid should be interpreted in this context.*
   c) *-Outcomes of WWE? L95 What outcomes?*
   d) *-Influence WWE in a specific way? L101. How?*
   e) *-sections L142.*
   f) *- should be obvious that induction or elective surgery are not spontaneous preterm births on L143-144.*
   g) *-Sentence “Other epilepsy core…” on line 162 if needed to address as a limitation should be in the discussion.*
   h) *-BMI abbreviation on L169 already defined on L88.*
   i) *-In Figure S1 total number of woman (observations) is a suboptimal measure for pattern of use as prevalence of WWE is not stable. Would be better to report per 100 / 1000 births to describe changes in prevalence of use.*
   j) *-Figure 1 consider rearrange so that there would be same scale on y-axis on figures within one row. P-values for trend women without epilepsy should be given in the Figure/Footnotes as interpretation of RRs is dependent on the rate in reference population.*
   k) *-Figure 3 should be figure 1, describing the setting in which study outcomes derive from. It is not necessary to give P-values for trend for WWE in Figure 3 either, could be in the footnote as for the reference group.*
   l) *-Lines 335-338, I don´t get the message.*
   m) *-Lines 373-378, I don´t get the message.*

**Response:**

We have rephrased our manuscript according to these suggestions:

a) This statement has been removed from the abstract. This aspect has been better explained in the introduction (page 3, line 91-92) and remains as a point of discussion (page 12-13, line 382-391).
b) We have rephrased the wording in these statements.
c) We have clarified the sentence in the revised manuscript.
d) We have clarified the sentence in the revised manuscript.
e) We have corrected the spelling.
f) We would argue that it is important to remind the reader of this exclusion in order to promote transparency of the methodology. We specifically highlight this because previous studies on WWE have not performed similar distinctions.
g) We have added the suggested limitation to the discussion (page 14, line 446-449). However, the core outcomes were considered while designing the study. Therefore, we do not think that this statement should be excluded from the methods section.
h) We have removed the definition.
i) We have changed the figure according to the suggestion from the reviewer. The figure now reports proportion of observed specific AEDs per WWE with AEDs. The figure remained essentially unchanged. This was also the case if the figure was presented as proportion of AEDs per all WWE.
j) The presented order of outcomes is reflected in the tables. Therefore, we would argue to maintain the order of outcomes as it stands in the figure in order to promote uniformity. However, we have revised the figure by enlarging the font size and enhancing numbers in bold along the y-axis. We have also included p-values for women without epilepsy in the footnotes.
k) The manuscript presents results in an order that we believe will contribute to an understanding of the findings. The most important findings are presented early in the results section. The order of figures follows the text and therefore also our narrative. We agree that maternal characteristics are essential to understand the results. However, in order to maintain flow in the text we would argue not to change the order of results or figures. We have removed p-values from figure 3 and added them as a footnote according to the suggestion from the reviewer.
l) We have rephrased this paragraph (page 11, line 334-337).
m) We have rephrased this paragraph (page 12, line 382-391).

**Reviewer #2:**

1. *Given the size of the population, some of the p values are an overestimate of the potential differences, particularly with comparisons with women without epilepsy. While implied by the comparisons with the general population, this is not a hypothesis testing study, and, since the entire population is used, not a sample, the differences in proportions is what you have regardless of the "p" value.*

**Response:**

Since numbers in some of our groups are relatively small, despite the fact that we capture all births in the population in the study-period, we need statistical inference measures to evaluate whether tendencies in the data are random fluctuations or may represent real underlying trends. P-values have been removed from tables where 95 % CI are presented because we want to put more emphasis on estimation rather than on formal hypothesis-testing. P-values are still important to evaluate interactions and changes over time.

2. *In figure 3, some of the graphs do not start at 0 for the abscissa giving a false impression of the degree of change to the casual reader. A break in the y axis line would seem appropriate.*

**Response:**

We have added a break on the y-axis in figure 3 where appropriate, and we have changed the remaining graphs to start at 0.

3. *It is my perception that a protective effect of folate for eclampsia has not been demonstrated in clinical trials. (line 342) Some additional discussion here may be appropriate. Has the dietary food chain been modified in Norway to enhance folate intake in the general population?*

**Response:**

We agree with the reviewer and have now removed the statement.

4. *I would say that while there may be more room for improvement in WWE, preconceptional counseling is important for all women (line 384 and 435).*

**Response:**

We have rephrased the remarked statements (page 13, line 399-400 and page 15, line 466-467).

All authors have approved the revised version of the manuscript.

Kim Christian Danielsson, Nils Erik Gilhus, Rolv Terje Lie, Ingrid Borthen, and Nils-Halvdan Morken.

Corresponding author: Kim Christian Danielsson, Department of Obstetrics and Gynecology,

Haukeland University Hospital, Postboks 1400, 5021 Bergen, Norway.

E-mail: kim.christian.danielsson@helse-bergen.no

**References:**

1.	Lisonkova S, Joseph KS. Incidence of preeclampsia: risk factors and outcomes associated with early- versus late-onset disease. American journal of obstetrics and gynecology. 2013;209(6):544 e1- e12.

2.	Lisonkova S, Sabr Y, Mayer C, Young C, Skoll A, Joseph KS. Maternal morbidity associated with early-onset and late-onset preeclampsia. Obstetrics and gynecology. 2014;124(4):771-81.

3.	Danielsson KC, Borthen I, Morken NH, Gilhus NE. Hypertensive pregnancy complications in women with epilepsy and antiepileptic drugs: a population-based cohort study of first pregnancies in Norway. BMJ open. 2018;8(4):e020998.

4.	Al-Zirqi I, Stray-Pedersen B, Forsen L, Kjersti Daltveit A, Vangen S, The NURg. Validation study of uterine rupture registration in the Medical Birth Registry of Norway. Acta obstetricia et gynecologica Scandinavica. 2013.

5.	Baghestan E, Bordahl PE, Rasmussen SA, Sande AK, Lyslo I, Solvang I. A validation of the diagnosis of obstetric sphincter tears in two Norwegian databases, the Medical Birth Registry and the Patient Administration System. Acta obstetricia et gynecologica Scandinavica. 2007;86(2):205-9.

6.	Thomsen LC, Klungsoyr K, Roten LT, Tappert C, Araya E, Baerheim G, et al. Validity of the diagnosis of pre-eclampsia in the Medical Birth Registry of Norway. Acta obstetricia et gynecologica Scandinavica. 2013.

7.	Skomsvoll J, Ostensen M, Baste V, Irgens L. Validity of a rheumatic disease diagnosis in the Medical Birth Registry of Norway. Acta obstetricia et gynecologica Scandinavica. 2002;81(9):831-4.

8.	Hoff JM, Daltveit AK, Gilhus NE. Myasthenia gravis in pregnancy and birth: identifying risk factors, optimising care. European journal of neurology : the official journal of the European Federation of Neurological Societies. 2007;14(1):38-43.

9.	Borthen I, Lossius P, Skjaerven R, Bergsjo P. Changes in frequency and indications for cesarean section in Norway 1967-1984. Acta obstetricia et gynecologica Scandinavica. 1989;68(7):589-93.

10.	Moth FN, Sebastian TR, Horn J, Rich-Edwards J, Romundstad PR, Asvold BO. Validity of a selection of pregnancy complications in the Medical Birth Registry of Norway. Acta obstetricia et gynecologica Scandinavica. 2016;95(5):519-27.
